# Prof. W. S. Armstrong

**Published:** 1867-10

**Authors:** 


### PROF. W. S. ARMSTRONG.

Prof. Armstrong, filling the chair of Anatomy in the Atlanta Medical College, sailed for Europe, on the 8th of last month, to be absent six months. He will visit London, Dublin and Paris, for the purpose of seeing their Schools and Hospitals, as a means of further prosecuting the study of his profession. Prof. A. is a young man of talents, and we doubt not will make a good use of his time.

